# Recurrent Potential G-Quadruplex Sequences in Archaeal Genomes

**DOI:** 10.3389/fmicb.2021.647851

**Published:** 2021-03-24

**Authors:** Galina V. Chashchina, Anna K. Shchyolkina, Simon V. Kolosov, Artemy D. Beniaminov, Dmitry N. Kaluzhny

**Affiliations:** ^1^Engelhardt Institute of Molecular Biology, Russian Academy of Sciences, Moscow, Russia; ^2^Moscow Institute of Physics and Technology, Dolgoprudny, Russia

**Keywords:** G-quadruplex, archaea, DNA, nuclease probing, circular dichroism, Methanomicrobiaceae

## Abstract

Evolutionary conservation or over-representation of the potential G-quadruplex sequences (PQS) in genomes are usually considered as a sign of the functional relevance of these sequences. However, uneven base distribution (GC-content) along the genome may along the genome may result in seeming abundance of PQSs over average in the genome. Apart from this, a number of other conserved functional signals that are encoded in the GC-rich genomic regions may inadvertently result in emergence of G-quadruplex compatible sequences. Here, we analyze the genomes of archaea focusing our search to repetitive PQS (rPQS) motifs within each organism. The probability of occurrence of several identical PQSs within a relatively short archaeal genome is low and, thus, the structure and genomic location of such rPQSs may become a direct indication of their functionality. We have found that the majority of the genomes of Methanomicrobiaceae family of archaea contained multiple copies of the interspersed highly similar PQSs. Short oligonucleotides corresponding to the rPQS formed the G-quadruplex (G4) structure in presence of potassium ions as demonstrated by circular dichroism (CD) and enzymatic probing. However, further analysis of the genomic context for the rPQS revealed a 10–12 nt cytosine-rich track adjacent to 3'-end of each rPQS. Synthetic DNA fragments that included the C-rich track tended to fold into alternative structures such as hairpin structure and antiparallel triplex that were in equilibrium with G4 structure depending on the presence of potassium ions in solution. Structural properties of the found repetitive sequences, their location in the genomes of archaea, and possible functions are discussed.

## Introduction

The functional role of G-quadruplexes (G4) and other non-canonical DNA structures in a wide range of organisms from humans to microbes and viruses is a subject of extensive investigation (Choi and Majima, 2011; Wang and Vasquez, 2014; Saranathan and Vivekanandan, 2019). Nucleotide sequence is a prior requirement for the formation of the G4 structures. Due to the roles of G4s in the regulation of diverse cellular processes, the identification of potential quadruplex sequences (PQS) in the genomes is an important task. Several algorithms are available for the prediction of the G-quadruplex-forming sequences, although each of them has certain advantages and disadvantages (Lombardi and Londoño-Vallejo, 2020). Analysis of nucleotide sequences for various organisms indicates that non-canonical structures can be potentially formed by DNA and RNA molecules in almost any organism, from humans to bacteria and viruses (Huppert and Balasubramanian, 2005; Maizels and Gray, 2013; Ding et al., 2018; Lavezzo et al., 2018; Brazda et al., 2020). Since the folding of G4 structures may create a certain obstacle for the processes such as replication and transcription, the living organism had to invent the mechanisms to overcome the hurdle or get rid of the genomic sequences with the capacity to form such alternative structures (Guo and Bartel, 2016; Sauer and Paeschke, 2017). These mechanisms are of special interest for extremophiles – the organisms adopted to survive at high salt conditions or unusual temperatures. In recent searches for PQS’s in the genomes of bacteria and archaea (Ding et al., 2018; Bartas et al., 2019; Brázda et al., 2020), a significant diversity in the distribution and density of such sequences was found within genomes of different organisms. In particular, the occurrence of PQS’s deviated considerably from random distribution in the genomes being over-represented in non-coding RNAs in archaeal but not in eubacterial genomes. This observation suggests the involvement of the G4 structure in the mechanisms of ncRNA function in archaea.

Our search for potential G4 sequences in the archaeal genomes shows that of PQS’s are extremely diverse in these organisms and the density of PQS’s significantly depends on the local GC composition. In this work, we have focused our attention on the study of repetitive PQS within each archaeal genome. The probability of occurrence of several identical PQS within a relatively short (a few Mb) archaeal genome is extremely low and, thus, the structure and genomic location of such a repetitive PQS sequences may provide a clue to their function.

## Materials and Methods

### DNA Sequence Analysis

Archaeal genomes (1,112 in total) with annotations (RefSeqs) were downloaded from the Assembly database of the National Center for Biotechnology Information https://www.ncbi.nlm.nih.gov/assembly/?term=txid2157[Organism:exp]. The search for PQS’s was performed with a modified Quadparser algorithm (Huppert and Balasubramanian, 2005). The algorithm was adopted to scan for G4 pattern containing G-runs of length 3–5 nucleotides and loops of 1–5 nucleotides (G_3-5_L_1–5_). These PQSs were searched with Python script in each genomic sequence joined with its reverse complement using regular expression with non-overlapping and non-greedy (lazy) matching “(G{3,5}?.{1,5}?G{3,5}?.{1,5}?G{3,5}?.{1,5}?G{3,5}?).” The total number of hits was divided by genome length to obtain the PQS density. For calculation of PQS density in a random sequence with fixed CG content, we generated five 10 Mb random strings, with a predetermined probability of each nucleotide according to GC content of the sequence. The same search algorithm was applied to search PQSs and calculate PQS densities in random strings. The average number of PQS on the five randomly generated 10 Mb string for each GC content and the standard deviation was calculated and plotted against the GC content.

The search for repeated PQS in each genome used a slightly different algorithm that allowed overlapping of PQSs. Then, from this redundant set of PQSs, we removed those that contained within itself a shorter variant, thus leaving only minimal or the shortest versions of PQSs. Each of this minimal PQS was tested for the presence of other copies in the genome. The PQSs that repeated four or more times in a genome (counting both the direct and its complementary sequence) were chosen for further study. Distances, feature names, strand orientation are collected for all analyzed PQSs and presented in Supplementary Table S1. Separately, a similar algorithm was applied for analyses of 20 RefSeq assembly genomes of Methanomicrobiaceae (txid2194) to search the repeated sequences within and between the species of this family. The raw data are deposited in Supplementary Table S2.

Alignment and phylogenetic analysis of 16S rRNA from Methanomicrobiaceae genomes was done with ClustalW (Larkin et al., 2007). Phylogenetic tree was built with Interactive Tree Of Life (iTOL) v4 (Letunic and Bork, 2019). Detection of the sequence motifs in the region that flanks PQS (100 nt upstream and downstream) was performed using GLAM2 Version 5.1.1 (Frith et al., 2008). The maximum number of columns aligned was set to 60; the initial number of key positions (aligned columns) in the alignment was 30. Ten alignment runs with 2,000 iterations were performed to obtain the highest score alignment. The other parameters were set by default in web server application.

### Enzymatic Probing

S1 nuclease was obtained from Thermo Fisher Scientific (United States). 5'-end fluorescein (FAM) labeled oligodeoxyribonucleotides (ODNs) were purchased from DNA-synthesis (Moscow, Russia; Table 1).

Probing with S1 nuclease was performed at 1 μM of ODN in 20 mM lithium acetate buffer pH 8.0 in the presence or absence of 100 mM KCl. The samples were renatured as described in the text. After renaturation, the buffer had been supplemented with 1 mM ZnSO_4_. Nuclease S1 (0.1 unit) was added and the samples were incubated for 3 min at 37°C. The reaction was stopped by ethanol precipitation. The G+A ladder was obtained by incubation of ODN in 50% formic acid for 10 min at room temperature. After treatment with formic acid the ODNs were ethanol precipitated, dissolved in the loading buffer containing 90% formamide, 30 mM sodium phosphate buffer (pH 8.0), 0.025% bromophenol blue, and 0.025% xylene cyanol. The digestion products were separated in a 16% denaturing gel containing 7 M urea at 20 V/cm for 40 min. The gel was visualized using a Typhoon FLA 9500 (United States) fluorescence scanner. S1 nuclease digestion was repeated at least twice for each solution condition and obtained consistent results.

**Table 1:** Nucleotide sequences of the synthetic oligodeoxyribonucleotides (ODNs).

ODNs	Sequence
GCA-L	FAM-CTGGGGGGTGGGGGCAGGGGAGGGGGCTCGCCCCCTCCCCGCA
GCA-S	FAM-CTGGGGGGTGGGGGCAGGGGAGGGGGCTC
ACC-L	FAM-CTGGGGGGTGGGGACCGGGGAGGGGGCTCGCCCCCTCCCCGCA
ACC-S	FAM-CTGGGGGGTGGGGACCGGGGAGGGGGCTC

### CD Spectroscopy

Oligodeoxyribonucleotides were dissolved in 20 mM lithium acetate buffer pH 8.0 at 1 μM concentration and annealed by heating to 90°C for 3 min and fast cooling to 20°C. Where indicated, KCl was added to 100 mM and the samples were renatured again by heating to 90°C and fast cooling. Circular dichroism (CD) spectra were performed using a Jasco J-715 spectropolarimeter (Japan) in a quartz cell of 10-mm optical path length and scanning speed 50 nm/min, with a response time 4 s. The spectra were recorded at 25°C in the wavelength range of 220–350 nm. Three scans were averaged.

## Results

**Figure 1:**
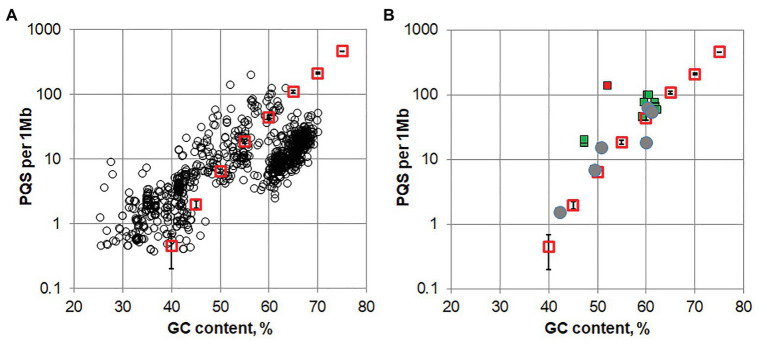
**(A)** The potential quadruplex sequences (PQS) density in archaeal genomes (black circles) and a random sequence (red squares, error bars shows SD in five 10 Mb replicates for random sequence) plotted against the G/C content of the genomes. **(B)** PQS density in genomes of Methanomicrobiaceae family. Filled green squares are genomes containing repetitive PQSs; gray circles present the genomes lacking repetitive PQS (rPQS); red square corresponds to Methanogenium cariaci JCM 10550 genome that contains about 100 repeated sequences of the pattern GnNiGn (where *n* > 7, *i* = 1 or 2).

A set of 1,112 archaeal genomic DNA sequences was downloaded from the Genome database of the National Center for Biotechnology Information. A search for G4 pattern (G_3-5_L_1–5_) revealed that 90% of the archaeal genomes contained one or several PQS, while about 10% of the analyzed genomes had no sequences matching the G4 pattern. The density of PQS for each archaeal genome was plotted against average GC content of the genome (black circles for each genomes sequence, Figure 1A). The average value of PQS density in a random nucleotide sequence (red open squares, SD of five independent 10 Mb random sequences shown with error bars) apparently an increases with its GC content. A strong correlation (Spearman’s correlation coefficient *r* = 0.83, *p* < 2e-16) between the PQS density in all archaea genomes and the average GC content of the genome is also visible. Relationship between PQS frequency and GC content in the genome for several groups of phylogenetically related archael species is shown on Supplementary Figure S1. One can also notice that the PQS’s are underrepresented in a considerable amount of genomes of Halobacteria class with high GC content (60–70%). The adaptation of genomes for high salt environment may result in selective sweep of some potential alternative nucleic acids structure formation, which stability strongly depends on salt concentration. On the contrary some enrichment in PQS is observed for genomes with low GC content, which is probably due to irregular GC distribution.

Next, we investigated the sequence diversity and prevalence of the identified PQSs. In particular, we were interested in those PQSs motifs that appear multiple times in an archaeal genome because chances of finding even two identical ca 15 nt long sequences in a several megabase genome are extremely low. The PQSs that repeated four or more times in a genome were selected for further analysis (Supplementary Table S1). It was not surprising that some thermophilic and halophilic archaea with high GC content contained homoguanine (G)n sequences that formally are PQS and potentially able to fold into G4 structure (Sengar et al., 2014). Some genomes comprised simple regular repeats, such as (GGGCT)n in *Candidatus Nitrocosmicus oleophilus* strain MY3, or (GGGCGA)n in *Halomicrobium mukohataei* strain ZPS1, or (GGGAA)n in *Halogeometricum borinquense* strain wsp4. These short tandem repeats theoretically match the G4 pattern; however, they appeared idiosyncratic for a particular organism and were not found in close organisms of the same family of archaea. All those short simple repeats were thus excluded from following analysis. Three evolutionary distant archaeabacteria *Methanogenium cariaci* JCM 10550, *Thermogymnomonas acidicola* JCM 13583, and *Thermocladium modestius* JCM 10088 had up to several 100 repeated sequences G_n_N_i_G_n_ (where *n* > 7, *i* = 1 or 2) interspersed in their genomes. Since these repeated sequences contained only two G-runs, they were also excluded in the current study.

**Figure 2:**
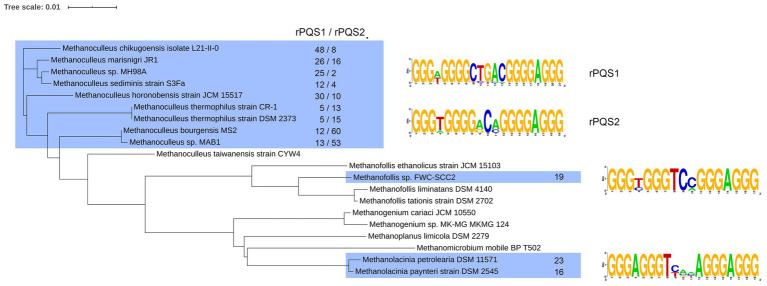
Phylogenic tree for *Methanomicrobiaceae* family based on alignment of 16S rRNA with ClustalW. The strains marked in blue contain the rPQS, which consensus sequences are shown on the left. Total number of each consensus rPQS is shown for each marked strain.

Another type of rPQS has been found in organisms belonging to the Methanomicrobiaceae family. The genomes of several bacteria from this family contained multiple copies of identical interspersed PQSs. We analyzed 20 Refseq assembly genomes Methanomicrobiaceae clade (taxid2194, NCBI) in search for such repetitive PQS. All 20 genomes contained PQSs, however, only 12 out of 20 analyzed genomes turned out to possessed highly repeated (Supplementary Table S2). The PQS density for the genomes of Methanomicrobiaceae is shown in Figure 1B. Apparently, the organisms containing rPQS (green squares) differ by slightly higher overall PQS density comparing to other bacteria that have only unique PQSs and lack rPQSs. All sequences and their copy numbers represented in the analyzed *Methanomicrobiaceae* genomes are shown in Supplementary Table S3. The four G-runs in rPQS were separated by a single non-G nucleotide between the 1st and the 2nd run, 3–5 nucleotides between 2nd and 3nd run and single A between 3nd and 4th G-run. The most obvious difference between these PQSs is the sequence of the central loop. *Methanoculleus* genomes have [CTGAC], [GCA], [ACC] or [ACA] central loop; *Methanolacinia* have [TCA(C|T)A] and [TT(C|G)AA]; *Methanofollis* sp. FWC-SCC2 has [TC(C|A)]. Some of the found sequences are repeated multiple times in only one species and are unique in this sense. Phylogenic analysis of *Methanomicrobiaceae* bactereria based on 16S ribosomal RNA has shown that some rPQSs were clustered to the nearest strains (Figure 2).

The search for the functional role of the found rPQS in the archaeal genomes was started with addressing PQS distribution in the genome relative to the origin of replication site. However, no regularity or common patterns were found. In Supplementary Figure S2, we show the distribution of PQS and rPQS along the genomes relative to the origin of replication taken from DoriC database (Luo et al., 2014; Luo and Gao, 2019) for some bacteria of Metanomicrobacteria family.

**Figure 3:**
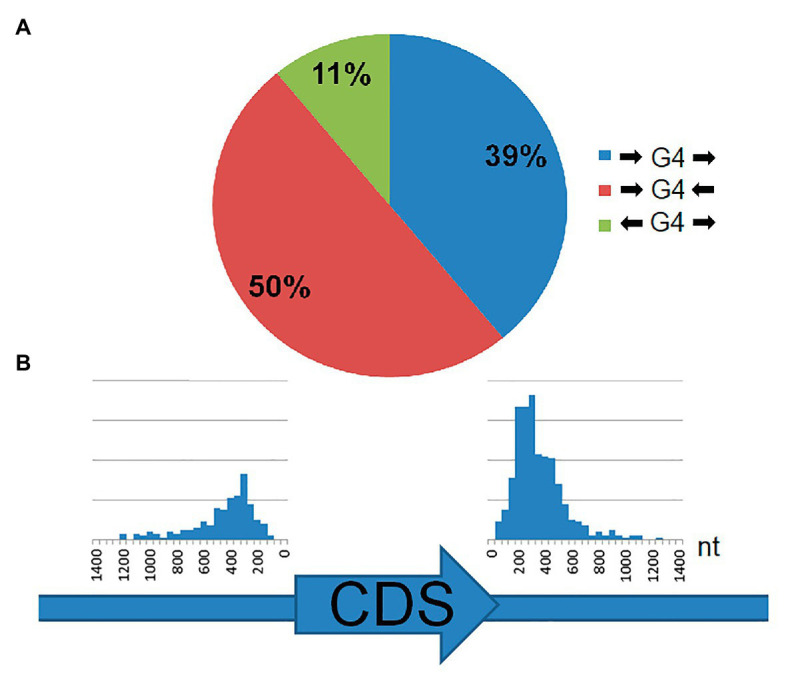
Location of rPQSs in 12 genomes of *Methanomicrobiaceae* family with respect to the two nearest genes. **(A)** Circular diagram of the rPQS distribution between the pairs of genes with head-to-head, tail-to-tail, and head-to-tail orientation. **(B)** The distance (in nucleotides) distribution between rPQS and the start of a CDS (left) or the end of a CDS (right).

Analysis of the genomic position of the rPQSs relative to coding region of the nearest genes revealed that all these sequences are located in intergenic regions. About half of rPQSs were found between two genes with tail-to-tail orientation, i.e., such rPQSs are close to the ends of the CDS of both genes (Figure 3A). Only 11% of rPQSs are located between the genes oriented in the opposite head to head direction. About 39% of the rPQSs appeared between a pair of genes with head to tail orientation. The above statistics was performed regardless of the sense or antisense orientation of the G-rich strand. Orientation of the PQS did not correlate with orientation of the nearby genes: the PQSs occurred in the sense and antisense strands in equal proportion. This latter observation may indicate that possible functionality of the putative G4 structures is linked to DNA rather than RNA. The average distance between a PQS and the start or the end of the closest coding sequences of the gene was about 300 nt (Figure 3B). That fact positions the majority of the PQSs close to the 3'- or 5'- untranslated regions of the transcripts of the considered genes. Thus these repeated sequences may potentially participate in initiation or termination of gene transcription. In a further search for functional role of the discovered rPQSs, we analyzed the functions of the nearby genes (summarized in Supplementary Table S4). No apparent correlation or overrepresentation of genes with certain functions was found. However, 9 out of 12 organisms contained rPQS close to DEAD/DEAH box helicase or related genes that may point to possible involvement of the rPQS in expression of Rho-helicases and transcription termination in archaea.

**Figure 4:**
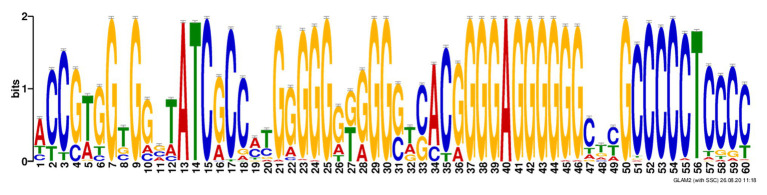
Alignment of the ±100 nucleotide context of the repetitive PQSs in the genomes of *Methanomicrobiaceae* family revealed a C-rich motif at 3'-end of the PQS. Discovered motif in *Methanomicrobiaceae’s* performed with GLAM2.

In search for additional sequence motifs in the 5'- and 3'- regions flanking the rPQSs, we discovered an interesting feature universal for all the studied sequences. Every rPQS in the genomes of *Methanomicrobiaceae* family was flanked at 3'-end by a C-rich sequence with a minimal motif C_3_TC_3_, which was partially complementary to the last two G-runs of the PQS (Figure 4). Thus, theoretically, all these sequences were able to form either G4 structure or an alternative GC-rich hairpin. In addition, interaction of the first two runs of the PQS with the hairpin may lead to formation of an intramolecular antiparallel triplex with a G-rich third strand. Obviously, folding of the GC-rich hairpin or triplex structure would compete with the formation of potential G-quadruplex structure.

**Figure 5:**
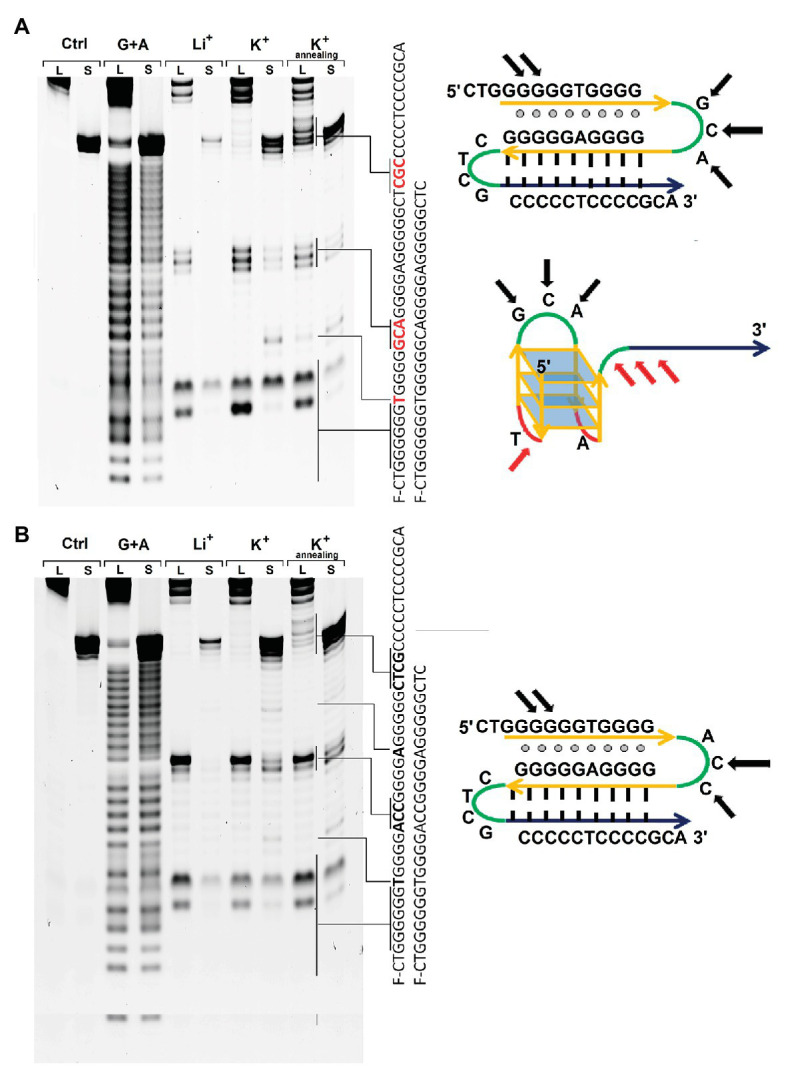
S1 nuclease probing of the structures formed by ODNs GCA-S and GCA-L **(A)** and ODNs ACC-S and ACC-L **(B)** in the presence of Li^+^ ions or K^+^ ions before and after renaturation. L – longer ODNs ACC-L or GCA-L, *S – shorter ODNs* GCA-S or ACC-S. Sequencing G+A lane was performed by treatment of the ODNs with formic acid. On the right – schematic depictions of the alternative structures that the longer ODNs GCA-L or ACC-L may adopt the intramolecular triplex and G4 folding. Black arrows indicate the S1 cleavage regions common for structures regardless solution conditions; red arrows show the bands that appear after renaturation of the long ODNs in presence of potassium ions.

The ability of the found rPQS to form alternative DNA structures in solution was tested using S1 nuclease and synthetic ODNs of two types. The first set included short ODNs GCA-S and ACC-S, which sequence was restricted to potential quadruplex sequence and did not include the C-rich tract (Table 1). The second set (ODNs: GCA-L and ACC-L) contained longer sequence spanning the rPQS and the C-rich region at 3'-end. In each pair, the sequences differed by the second loop (GCA or ACC) in the PQS, being most representative and abundant sequence variants among the found rPQSs in the genomes of *Methanomicrobiaceae* family (Figure 5).

Every ODN was probed with nuclease S1 in three different conditions. First, the ODN was annealed in 20 mM lithium acetate buffer pH 8.0 by heating to 90°C and snap cooling to 20°C. Second, potassium chloride was added to this sample and incubated 10 min at room temperature. Third, the latter sample was renatured by heating to 90°C and fast cooling to 20°C.

The S1 cleavage pattern for short ODN GCA-S or ACC-S was in a good agreement with of G4 DNA structure in the presence of potassium ions (Figure 5). The cleavages of the first (single T) and second loop (GCA or ACC) appeared after addition of KCl in to the solution. The third loop (A) was weakly cleaved in ACC-S and remained protected from S1 cleavage in GCA-S that may indicate the low accessibility of this nucleotide for the nuclease or some steric hindrance. Instead, S1 nuclease cleaves a guanine in the center of the first 6-guanine run that may assume some sliding of such a long G-run and alternative involvement of guanines in folding of G4 structure or admixture some other structures under these conditions in solution.

The pattern of the S1 cleavage for the full-length ODNs GCA-L and ACC-L is in agreement with initial formation of a hairpin structure with a protected CTCG loop. Guanines at the 5' end of the first guanine run were also accessible to nuclease possibly due to the dangling of terminal nucleotides. However, most of the guanines before the GCA loop were protected from the action of S1 nuclease, which indicates a possible interaction of the G-rich region with the GC-hairpin structure and antiparallel triplex structure formation.

The probing pattern of the fragments GCA-L or ACC-L does not alter significantly upon addition of potassium ions, as it happened for the short ODNs GCA-S or ACC-S implying different structures for long and short ODNs under these conditions. Further annealing of the long ODN GCA-L in the presence of potassium ions significantly changes the cleavage pattern. A cleavage between the first two blocks of guanines begins to appear, similar to that observed for a short fragment capable of forming only the G4 structure. Thus, addition of potassium is not sufficient for changing the structure, but temperature elevation is required to achieve restructuring. ACC-L does not change cleavage pattern even after annealing with potassium ions. Probably central loop sequence is more favorable for hairpin formation then G4 structure.

**Figure 6:**
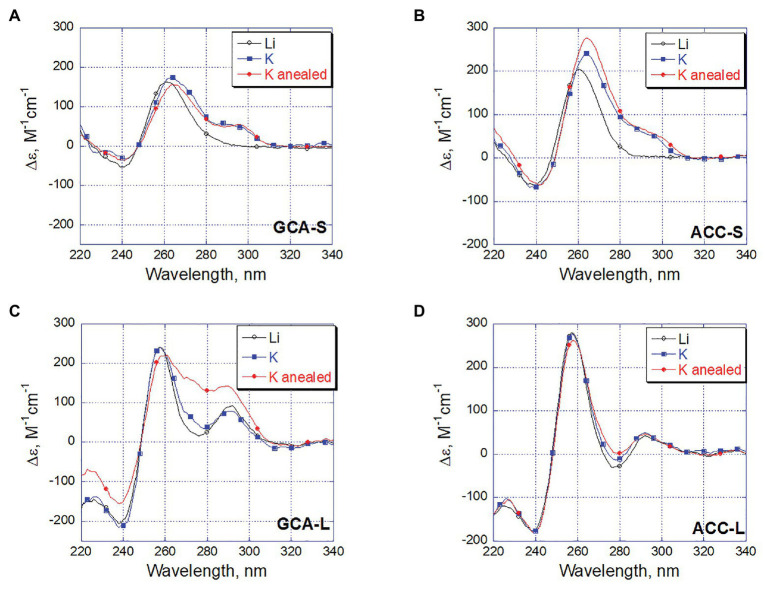
Circular dichroism (CD) spectral changes reflects potassium dependent DNA refolding. The spectra were recorded for oligonucleotides GCA-S (A), ACC-S (B), GCA-L (C), and ACC-L (D) at 1 µM concentration in 20 mM LiAc buffer pH 8.0 (black). The same sample after addition of 100 mM KCl and incubation 10 min at 20°C (blue) and subsequent fast annealing of the final solution (red).

Changes in CD spectra reflect the differences in conformation of DNA in solution. Thus, we recorded CD spectra of the four ODNs in the same three solution conditions that were used for S1 nuclease probing. The CD spectra of the short ODNs ACC-S and GCA-S in the presence of lithium ions had remarkable positive band at 260 nm, which most likely reflects residual stacking of guanine repeats. When potassium ions were added, the maximum shifted to 265 nm and another positive band appeared at 295 nm (Figures 6A,B). Such changes in CD spectra argue for formation of the G4 structure in which guanines are in both anti- and syn-conformation. Annealing of the short ODNs in the presence of potassium ions does not significantly change the spectral characteristics of the structure, which means that the G4 conformation formed simply by the addition of potassium ions is most preferable for the short ODNs.

Circular dichroism spectra of the long oligonucleotides in the presence of Li^+^ and K^+^ (black and blue spectra of ACC-L and GCA-L, Figures 6C,D) are the characteristics of the oligonucleotide fold comprising all three strands (Holder et al., 2015). Such a structure formed already in the Li^+^-containing buffer without the addition of K^+^ ions. It is known that lithium ions do not prevent the formation of a DNA triplex (Floris et al., 1999).

Dramatic conformational changes observed by CD were noted for oligonucleotide GCA-L after annealing in the presence of potassium ions (Figure 6C, GCA-L, red curve). An increase in the positive band in the 270–280 nm region can reflect the formation of a G4 structure accompanied by unweaving of the C-rich filament from the hairpin part. As noted earlier, the C-rich strand involved in Watson-Crick pairing and therefore protected from S1 nuclease, dissociates from the complementary sequence in the course of annealing.

Interestingly, neither the CD spectra nor the probing experiments showed a considerable conformational changes in the ACC-L oligonucleotide upon annealing (Figure 6D, ACC-L). Probably, a nature and conformation of a loop sequence may contribute to a conformational equilibrium between the oligonucleotide structures studied.

**Figure 7:**
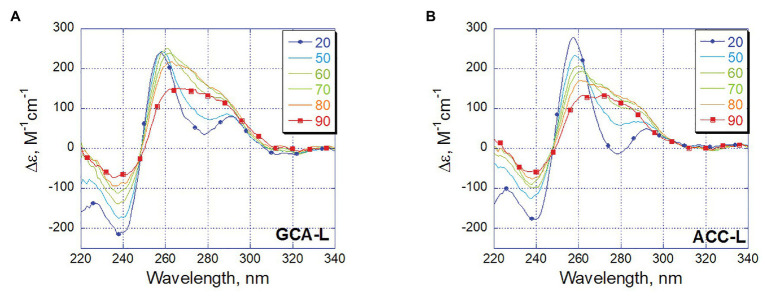
Temperature dependence of CD spectra for long oligonucleotides GCA-L **(A)** and ACC-L **(B)**. Temperature color code shown on insert from blue (20°C) to red (90°C).

Let us trace how the conformation of long oligonucleotides GCA-L and ACC-L changes with increasing temperature (Figure 7). The blue spectra correspond to an ordered triplex structure at 20°C, and the red spectra correspond to a completely disordered oligonucleotide at 90°C. So, a positive band appears in the broad region around 280 nm, which corresponds to the appearance of various non-interacting bases, while changes around 260 nm speak mainly of the interaction of guanines. Following the changes in CD in 260 and 280 nm with increasing temperature, we see an almost synbatic change in both bands in the GCA-L, which indicates the parallel rearrangement of the C-rich strand and the G-rich part. Unlike this, the band around 260 nm of ACC-L retains its intensity even with almost complete saturation of the positive band at 60°C (280 nm). Thus, judging by CD, some conformational rearrangements of G-rich part, namely, to G-quadruplex may take place in this structure upon the triplex melting and C-rich strand daggling approximately at 60°C. At higher temperatures, above 60°C, the oligonucleotides melt.

## Discussion

Earlier, potential quadruplex sequences in microorganisms have been studied in terms of their location relative to genomic features. It was found that the frequency of occurrence for some bacteria and archaea differs from random in certain non-coding regions of the genome, such as promoter regions, genes for non-coding RNA, etc. (Ding et al., 2018; Bartas et al., 2019; Brazda et al., 2020; Brázda et al., 2020).

In this work, for the first time, we performed the search for repeated regions in the genomic DNA of archaea, potentially capable of forming G4 structures. Some analyzed genomes comprised simple regular repeats that were individual for a particular strain and did not have wider distribution in phylogenetically close archaea. Among analyzed archaeal genomes, only the methanogen family strains were found to contain abundant repetitive PQS, and it was these rPQS that were accompanied by a cytosine rich region at the 3' end of the quadruplex sequence. All these rPQSs were located in intergenic regions. Analysis of the conformation formed by this nucleotide sequence indicated the formation of a triplex structure under certain condition in solution, with a stable hairpin region formed by GC pairs and a guanine-rich antiparallel third strand. Melting and subsequent annealing of this sequence in the presence of potassium ions promotes the conformational transition of the guanine-rich region into a quadruplex structure with unweaving of the stable GC hairpin. The majority of the found sequences were located in the non-coding regions of the genome close to the end or start of the coding sequence. It is tempting to assume some involvement of these sequences in regulation of transcription initiation or termination given the location of the motif in the genome and its structural lability. These archaeal rPQSs have not been previously found and analyzed, when the search was performed with the G-hunter algorithm (Brázda et al., 2020) since the algorithm is aimed at finding GC asymmetry in the genomic sequence. Due to the presence of the downstream conserved C-rich tract, these archaeal rPQS would receive a low score according to G-hunter algorithm. Interestingly, similar repeating motifs in which PQS are adjacent to a partially complementary site are found in bacteria. When searching for PQS in the *Escherichia coli* genome (Kaplan et al., 2016), guanine-rich regions adjacent to the cytosine region were found. However, in contrast to our finding, the cytosine-rich site is located at the 5' edge of the PQS. Formation of the triplex structure by the repetitive *E. coli* sequence was also proposed (Holder et al., 2015). The conformational transformation of such regions in DNA *E. coli* was associated with the onset of genomic instability (Hoyne and Maher, 2002; Holder et al., 2015). It is worth noting that other bacteria *Deinococcus radiodurans*, for which PQS have been found to be important, have similar repeating sequences (see Supplementary Table S5). They are located in non-coding regions of genes, in the promoter regions of genes responsible for repair, as noted in Ding et al. (2018). In addition, a large number PQS was found at the ends of the coding regions. The PQSs in the genomes of *D. radiodurans* or *E. coli* had a conserved cytosine rich region directly adjacent to 5'-end of the PQSs. Thus the difference between the bacterial PQSs and those found by us lies in the location of the cytosine sequence relative to the PQS. However, from a structural point of view, both sequences may adopt a triplex fold with a stable GC duplex supplemented by a third guanine-rich strand in an antiparallel orientation. Thus, the potentially quadruplex sequences found by us are similar in their conformational potential to non-coding regions in bacterial genomes and possibly have a similar origin or function.

## Conclusion

We found a recurrent sequence motif in the regulatory regions of genes in several genomes of the methane producing archaea. The motif comprises a PQS and downstream C-rich sequence partially complementary to the PQS. In solution, the single-stranded DNA fragments corresponding to this motif were able to form either G4 structures or GC-rich hairpin or an intramolecular antiparallel triplex with a G-rich third strand as confirmed by circular dichroism and nuclease S1 probing. Thus, the ability of this highly repetitive sequence to form several alternative DNA structures and its predominant location in the noncoding regions of archaeal genomes may suggest a regulatory role of these sequences in certain cellular processes.

## Data Availability Statement

Publicly available datasets were analyzed in this study. This data can be found at: https://www.ncbi.nlm.nih.gov/assembly/?term=txid2157[Organism:exp].

## Author Contributions

DK, AB, and AS: conception and design of the work and critical revision of the article. GC and SK: data collection. DK and SK: computation. GC, AB, AS, and DK: data analysis and interpretation. DK and AB: drafting the article. GC, AS, SK, AB, and DK: final approval of the version to be published. All authors contributed to the article and approved the submitted version.

### Conflict of Interest

The authors declare that the research was conducted in the absence of any commercial or financial relationships that could be construed as a potential conflict of interest.
