# Supplementary material for: Recurrent Potential G-Quadruplex Sequences in Archaeal Genomes
Source: Front Microbiol. 2021 Mar 24;12:647851. doi: 10.3389/fmicb.2021.647851 (PMC8044849; doi:10.3389/fmicb.2021.647851)
Supplement: Supplementary file 1 [file Data_Sheet_1.docx]

**Supplementary material**

**Recurrent potential G-quadruplex sequences in archaeal genomes**

Chashchina GV, Shchyolkina AK, Kolosov SV, Beniaminov AD, Kaluzhny DN

**Figure S1.** Relationship between PQS frequency and GC content in the genome for some groups of phylogenetically related species is shown in different colors. Spearman's rank correlation coefficient for each subset of genomes is given on the right.

**Figure S2.** The amount of PQSs (red points) or rPQSs (blue points) in the genomes do not correlate with the distance to the replication origin or GC content (averaged in a 200bp window, green line). Replication origin is shown with the blue flag.
